# Dr. Eleanor McAllister

**Published:** 1890-10

**Authors:** 


DR. ELEANOR MCALLISTER, of Newburgh, N. Y., has been appointed a member of the medical staff at the State Hospital, in this city, and has already entered upon her duties. She is a graduate of the Syracuse Medical College, being second in standing in her class. After several months of special work in gynecology, she accepted a position on the staff at the Willard State Hospital to fill a temporary vacancy, remaining there about six months, and giving abundant satisfaction to the managers and superintendent. She comes most highly recommended, and is an acquisition to our ranks, whom the profession of this city will heartily welcome.

